# Analysis of Bacterial Communities in Partial Nitritation and Conventional Nitrification Systems for Nitrogen Removal

**DOI:** 10.1038/s41598-018-30532-4

**Published:** 2018-08-28

**Authors:** Zhirui Zhao, Jinxue Luo, Bo Jin, Jiayao Zhang, Bin Li, Bin Ma, Xiaoyu An, Shujun Zhang, Baoqing Shan

**Affiliations:** 1 Research Center for Eco-Environmental Sciences, Chinese Academy of Sciences, Beijing, 100085 China; 2 College of Water Resources and Environment, Hebei Geo University, Shijiazhuang, 050031 China; 3 Beijing Drainage Group Corporation Limited, Beijing, 100124 China; 4 School of Chemical Engineering, The University of Adelaide, Adelaide, SA 5095 Australia

## Abstract

This work studied the microbial community in partial nitritation and complete nitrification processes, which were applied to treat the low Carbon Nitrogen ratio wastewater. The phospholipid fatty acid and quantitative PCR analysis showed that the sludge circulating ratio of 75% resulted in a good microbial growth and a higher abundance of ammonia oxidizing bacteria relative to the nitrite oxidizing bacteria. The Betaproteobacteria were observed to compose the most abundant sludge bacterial groups in the two processes, based on phylogenetic analysis. The phylogenetic analysis of both 16S rRNA and *amo*A gene indicated that the *Nitrosomonas* sp. were the dominant ammonia oxidizing bacteria in the partial nitritation process. The relative abundance of nitrite oxidizing bacteria, such as *Nitrobacter* sp. and *Nitrospira* sp., were significantly lower in the partial nitritation system over the complete nitrification system. The abundance of Planctomycetes was higher in the partial nitritation process, indicating the anammox reaction occurred in the partial nitritation system. These results suggested the nitrite accumulation rate of circulating ratios 75% was the highest, with an average of 92%,and a possibility to treat the low Carbon Nitrogen ratio wastewater using the partial nitritation/anammox process.

## Introduction

Conventional nitrogen removal by activated sludge in wastewater treatment is performed in a nitrification/denitrification process^1^. In conventional nitrogen removal process, the first step is to oxidize ammonia to nitrite (nitritation) by ammonia oxidizing bacteria (AOB), followed by oxidizing nitrite to nitrate (nitratation) by nitrite oxidizing bacteria (NOB), where oxygen (O_2_) is used as the electron acceptor, and the first two processes are nitrification. The last step, the denitrifying bacteria convert nitrate to nitrogen under anaerobic conditions^2^. Thus, a conventional nitrification/denitrification process needs three steps to remove nitrogen. In the denitrification^3^, nitrate and nitrite are reduced to gaseous nitrogen (N_2_) by heterotrophic microorganisms under anaerobic or anoxic conditions^4^. The discovery of anaerobic oxidation ammonium (anammox) and nitrite reduction with N_2_ as the end product in natural and man-made ecosystems challenged conventional process and has been recognized as an attractive alternative nitrogen removal process^5^. In comparison with the conventional nitrification/denitrification process, the partial nitritation/anammox process requires low oxygen consumption and produces low sludge biomass^5,6^.

In a partial nitritation/anammox process, it is crucial to suspend the nitrification reaction at the nitritation step, where the nitrite is produced and accumulated rather than oxidized further to nitrate in the nitritation step. Inhibition of NOB was critical in maintaining the nitritation performance in the nitritation step, as the existence of the NOB can oxidize nitrite to nitrate and converts the partial nitritation process to a complete nitrification process^7,8^. It was reported that the ratio of AOB/NOB for the nitritation process varied in a range of 2.0–3.5 under the suitable conditions^9^. Many studies have investigated the bacterial community in the nitrification process. In a complete nitrification process, the dominant bacteria of AOB and NOB were reported to be *Nitrosomonas* sp. and *Nitrobacter* sp. in many reported studies^10^, while *Nitrosospira* sp. and *Nitrospira* sp. were discovered as the main genera of AOB and NOB in a case study^11^. Therefore, there seems to be no consensus for the dominant AOB and NOB in the nitrification process. Moreover, the C/N ratio also plays an important role in affecting the dominant nitrifying bacterial compositions. When the C/N ratio was 0.5–2, the nitrifying bacterial community was dominated by *Nitrosomonas* sp. and *Nitrobacter* sp.^12^. Nevertheless, when the C/N ration increased to 4–15, the dominant nitrifying bacteria became *Pseudomonas* sp., *Acidovorax* sp. and *Comamonas* sp. Thus, more works are required to elucidate the microbial community in the nitritation and nitratation processes.

The objective of this study is to reveal the bacterial communities in the partial nitritation and complete nitrification systems in the treatment of low C/N ratio wastewater. The effects of sludge circulating ratio on the nitrifying bacterial compositions were studied. The differences of bacterial community in the partial nitritation and complete nitrification reactors were analysed and compared.

## Results and Discussion

DO and pH were measured at each sampling by the DO and pH meters. When the sludge circulating ratio were different, the nitrite accumulation rate of 25% was the lowest, with an average of 73%. The nitrite accumulation rate of circulating ratios of 50% and 100% were corresponding to the average cumulative rate of nitrite were 87% and 81%, respectively. The nitrite accumulation rate of 75% was the highest, with an average of 92%, so the reflux ratio of 75% was chosen as the study goal. At the circulating ratios of 75%, the concentrations of NH_4_^+^-N, NO_2_-N, and NO_3_-N are 29.1 mg/L, 10.6 mg/L and 1.3 mg/L in the first aerobic compartment, respectively. The nitrite accumulation rate was 90%. During the process of advancing the liquid mixture along the compartments, NH_4_^+^-N gradually decreased, and NO_2_-N and NO_3_-N gradually increased. When the mixture reached the last chamber of the reactor, the concentrations of NH_4_^+^-N, NO_2_-N, and NO_3_-N were 13.9 mg/L, 22.6 mg/L, and 2.1 mg/L, respectively. The nitrite accumulation rate was 92%. That nitrite accumulation rate was calculated through Nitrite accumulation rate = Transformed (nitrite)/ransformed (nitrite + nitrate) × 100%.

**Figure 1:**
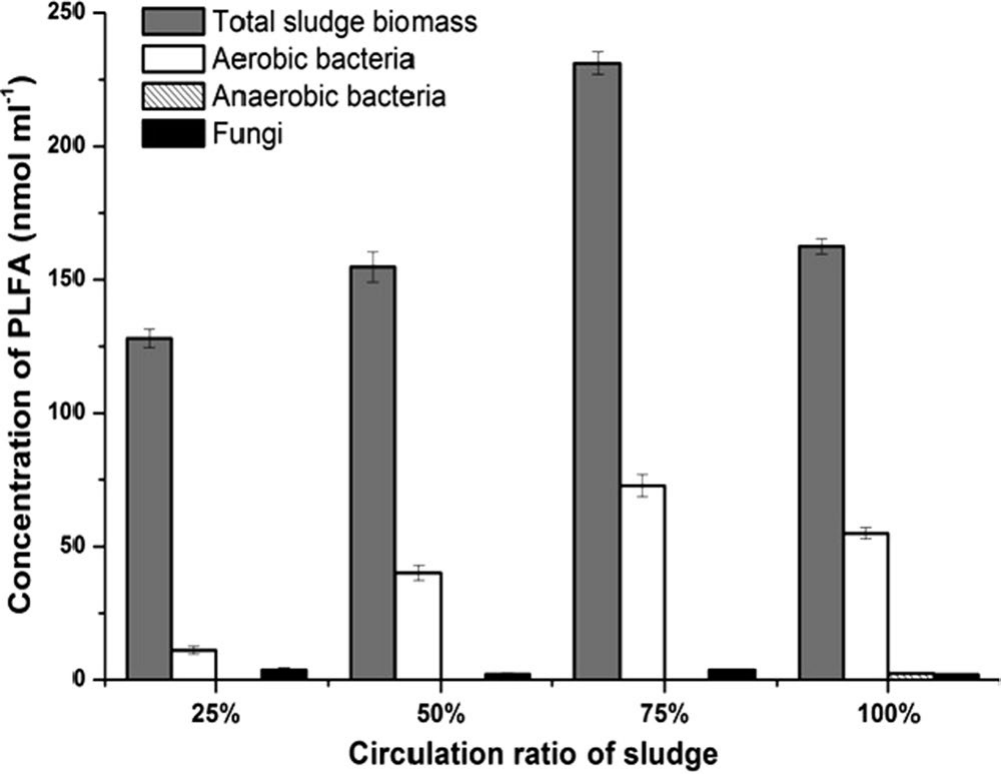
The concentrations of phospholipid fatty acids (PLFA) in sludge during the partial nitritation process. The error bars are the standard deviations (n = 3).

The biomass of microorganisms grown in sludge was indicated by the phospholipid fatty acids (PLFA) when the partial nitritation process was performed. It was found that the total PLFA in sludge was changed under different sludge circulating ratios (Fig. 1). The aerobic bacteria and fungi existed in sludge under the sludge circulating ratios from 25% to 100%, while the anaerobic bacteria only appeared in sludge when the sludge circulating ratio was adjusted to 100%. Moreover, the aerobic bacteria were the dominant microbial group in sludge at different sludge circulating ratios (11.1–72.8 nmol/ml), followed by the fungi (2.0–3.8 nmol/ml) and the anaerobic microorganisms (0–2.4 nmol/ml) (Fig. 1). It was further found that the sludge circulating ratio of 75% resulted in a highest quantity of nitrite and aerobic bacteria, indicating the optimal circulating ratio of sludge was 75%.

**Figure 2:**
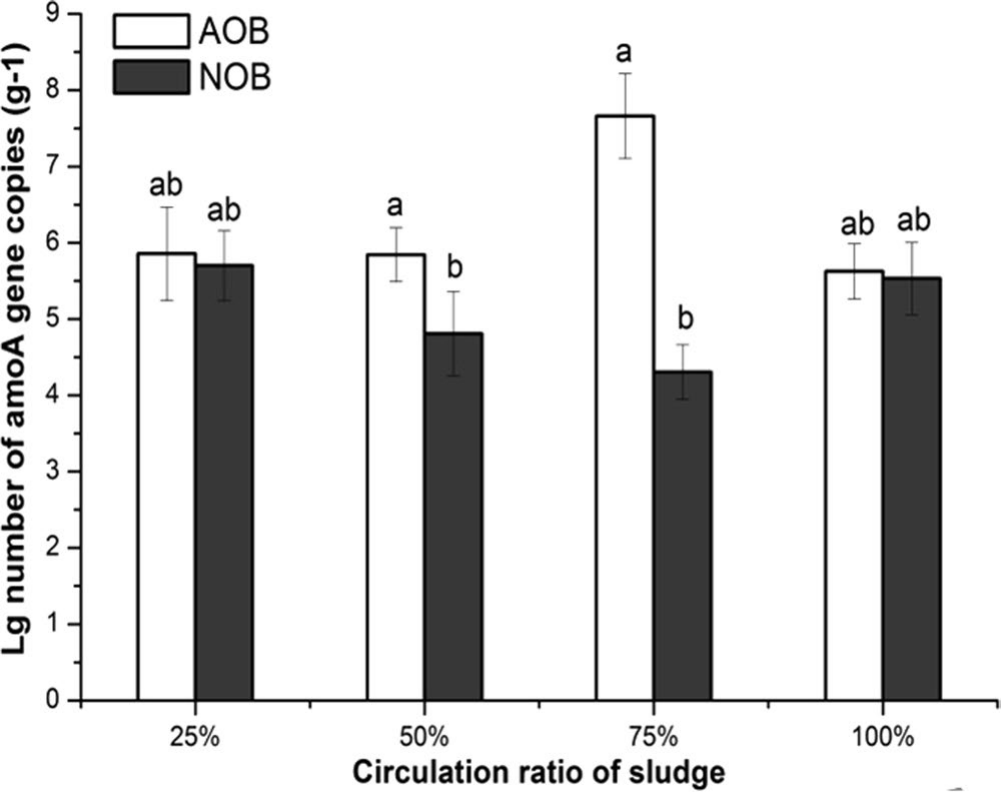
The quantity of *amo*A gene for AOB and NOB in activated sludge in the partial nitritation process. The number of the *amo*A gene copies was normalized as the Log10 number per 1 gram sludge. The error bars are the standard deviations (n = 3). The columns under different letters (ab, a, b) are significantly different at p < 0.05 (t test).

The amounts of AOB and NOB were quantified in the partial nitritation process through qPCR. It was observed that the quantities of AOB and NOB were almost the same when the sludge circulating ratios were 25% and 100% (Fig. 2). However, when the reactor was operated under the circulating ratios of 50% and 75%, AOB were found to be higher than NOB in activated sludge. When the circulating ratio was 50%, AOB and NOB were determined multiple to 1.0 × 10^5^/g and 1.0 × 10^4^/g, respectively. The result was observed for the circulating ratio of 75%, where AOB and NOB were multiple to 1.0 × 10^7^/g and 1.0 × 10^4^/g, respectively. Furthermore, the abundance of AOB in sludge was higher under the circulating ratio of 75% in comparison with the quantity under the circulating ratio of 50%. Under the circulating ratios 75% conditions, AOB doubling time is less than that of NOB, which is more conducive to the operation of a partial nitritation process. The nitrite accumulation rate of circulating ratios 75% was the highest, with an average of 92%, and the amounts of AOB was the same result from the Fig. 2. These results suggested that the optimal circulating ratio for partial nitritation was 75%, which was consistent with the conclusion from PFLA analysis in this experiment (section 3.1). Thus, this could be concluded that the sludge circulating ratio of 75% favoured the growth of AOB rather than the NOB under oxygen-limiting conditions.

**Figure 3:**
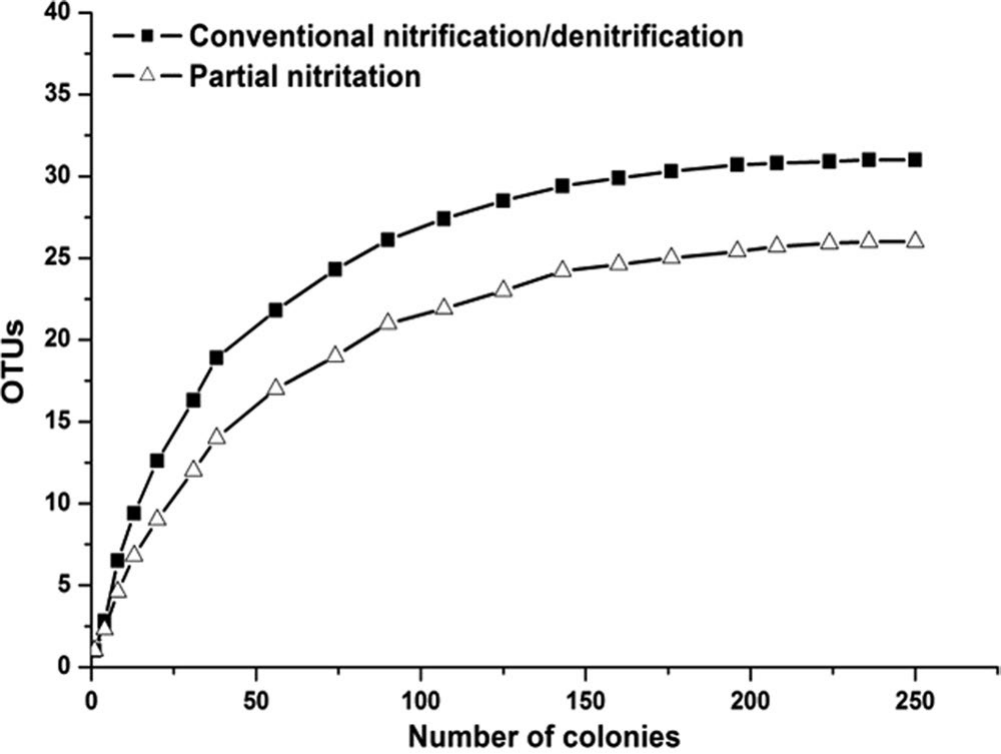
Rarefaction analysis of 16S rRNA gene clone library. Which the rarefaction using Rarefactwin Version 1.3 software.

A 16S rRNA clone library was constructed for the complete nitrification system. A total of 26 OTUs were collected from 260 clones based on the restriction fragment length polymorphism(RFLP) screening. The density of the clone library was evaluated statistically by the rarefaction curve (Fig. 3), which tended to approach a saturation plateau phase as the increase of the colony numbers. These results indicated that the majority of the operational taxonomic units(OTUs) in the complete nitrification process were included in this clone library. GenBank accession numbers were assigned to the 16S rRNA gene sequences of the isolates, which were from HQ343206 to HQ343226.

**Figure 4:**
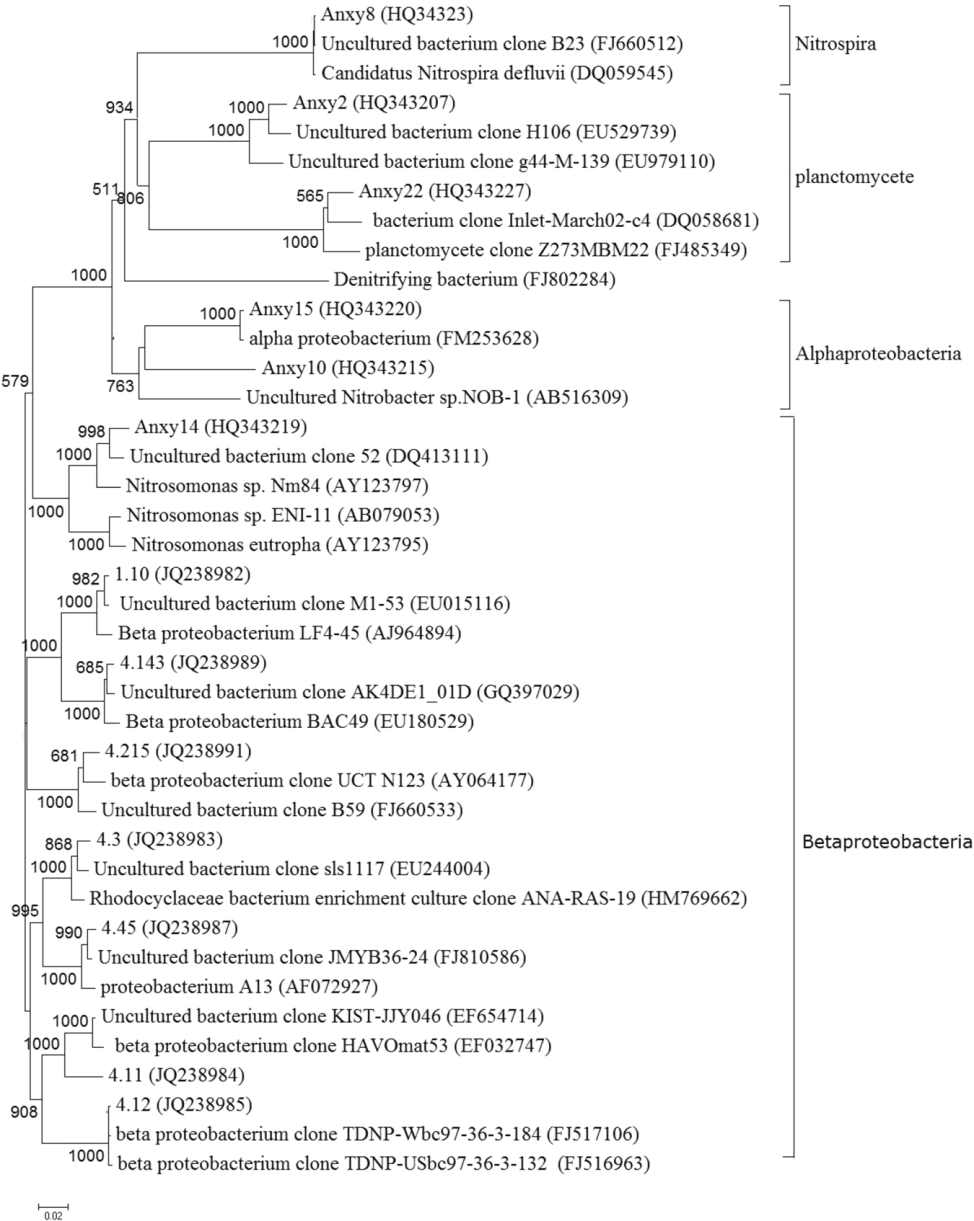
The phylogenetic tree of the 16S rRNA sequences in the complete nitrification process. The construction of the phylogenetic tree was based on the Neighbour-joining method. The scale bar indicates two changes per 1000 nucleic acid positions.

A total of 11 Classes of bacteria were assigned to these 26 OTUs. Among them, Beta-proteobacteria were the most dominant microbial groups and represented by 10 OTUs, which accounted for 36.4% of total OTUs. Among these 10 OTUs, the OTU Anxy14 had a close relationship with the AOB, *Nitrosomonas* sp., and the similarity is 97%, indicating that they were involved in the ammonia oxidation (Fig. 4). Two groups of nitrite oxidizing bacteria were detected in the complete nitrification process. The first group included 2 OTUs under the Alpha-proteobacteria (OTU Anxy15 and OTU Anxy10, which two similarities are all 99%), which were very similar to the *Nitrobacter* sp. Another group was represented by the OTU Anxy8, which was similar to the *Nitrospira* sp. These two groups of nitrite-oxidizing bacteria accounted for 18% of the total bacterial OTUs. In addition, other dominant bacterial groups were determined as Gamma-proteobacteria (13.8%), Chlorobi (13.4%), Delta-proteobacteria (13.5%) and Planctomycetes (2.3%) (Fig. 4).

Another 16S rRNA gene clone library was constructed for the partial nitritation system. 31 OTUs were acquired from 249 colonies based on RFLP analysis. GenBank accession numbers were assigned to the 16S rRNA gene sequences of the isolates, which were from HQ014631 to HQ014661.

**Figure 5:**
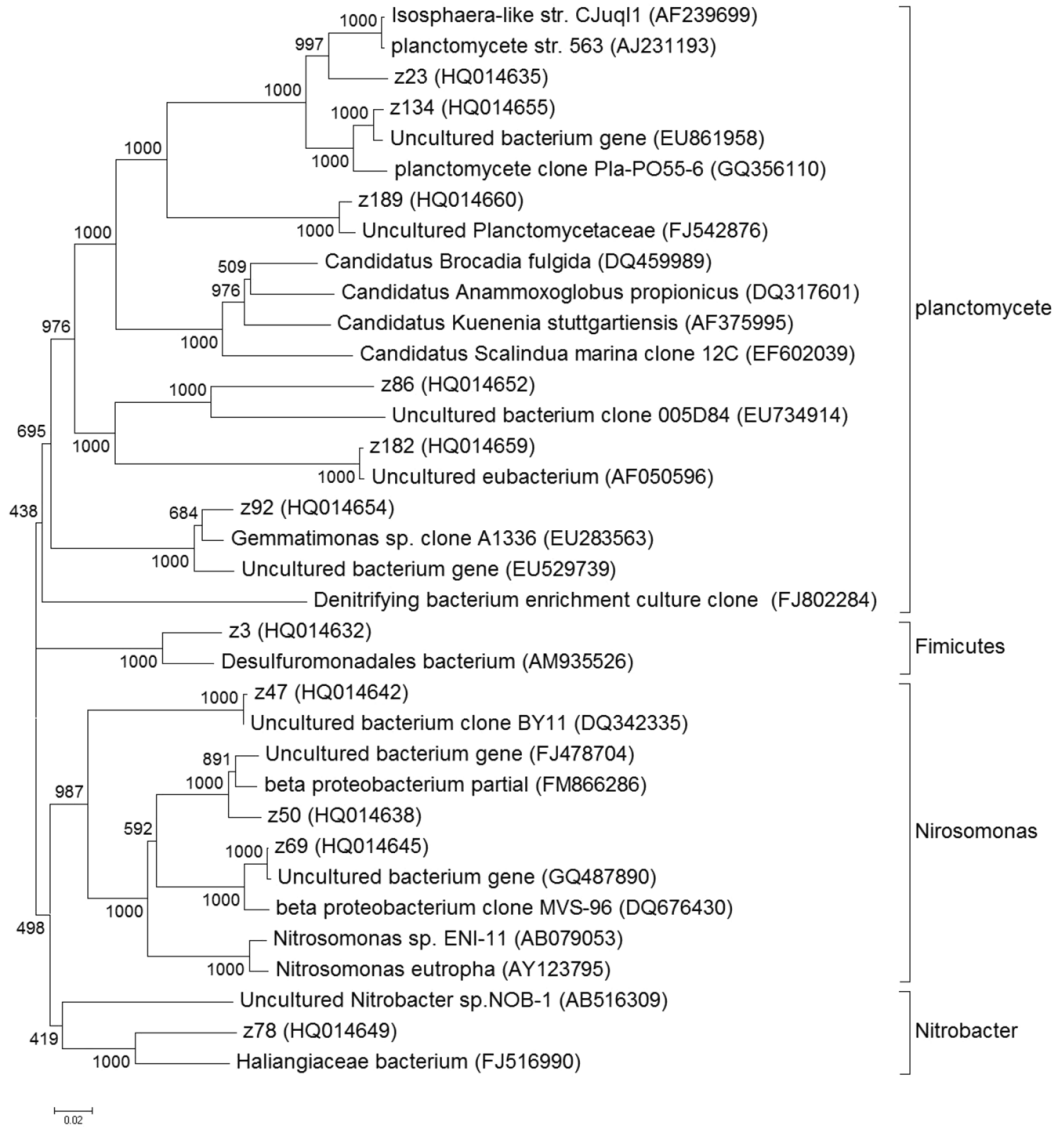
The phylogenetic tree of the 16S rRNA sequences in the partial nitritation process. The constrction of the phylogenetic tree was based on the Neighbour-joining method. The scale bar indicates two changes per 1000 nucleic acid positions.

Based on the phylogenetic analysis of the partial nitritation bacterial community (Fig. 5), it was found that the Beta-proteobacteria were the most abundant microbial group in the partial nitritation process and the complete nitrification process in this study (section 3.3). Similar results were also reported in the previous studies, where the Beta-proteobacteria were the dominant microbial group in the ammonia oxidizing process under both the low and high oxygen environment^13^. The Beta-proteobacteria in the partial nitritation process were represented by 3 OTUs, which accounted for 28.1% of the total OTUs. It was further noted that these 3 OTUs had a close relationship with the ammonia oxidizing bacteria *Nitrosomonas* sp., and the similarities of z47, z50,z69 were 99.7%, 97%, 97.5, respectively. that indicating they may play a role in the oxidation of ammonia to nitrite. Some studies reported that *Nitrosomonas* sp. under Beta-proteobacteria were the most important AOB in wastewater treatment systems^14^. This conclusion was supported by the results presented in our study. Other abundant bacterial groups included Chloroflexi, Bacteroidetes and Planctomycetes, which had the relative abundance of 28%, 22.9% and 9.6%, respectively, in the bacterial community of partial nitritation. The Alpha-proteobacteria were also detected in the partial nitritation process and were represented by OTU z78. The relative abundance of Alpha-proteobacteria was lower (2.0%) than Beta-proteobacteria (28.1%) and Planctomycetes (9.6%). It was noted that the OTU z78 has a close relationship with the uncultured *Nitrobacter* sp. NOB-1, and the similarity is 92%, indicating OTU z78 may be involved in the oxidization of nitrite to nitrate.

**Figure 6:**
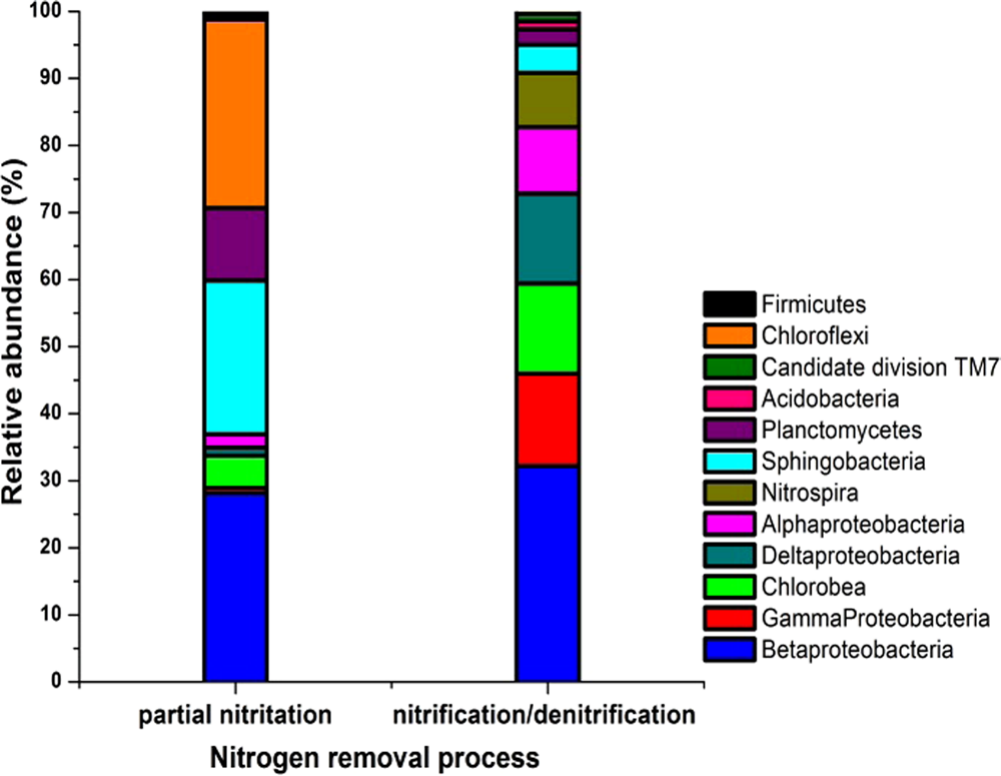
The bacterial community in activated sludge in the partial nitritation process and complete nitrification process.

In comparison with the complete nitrification process (Fig. 6), 3 groups of bacteria were enriched in the partial nitritation process, including Sphingobacteria, Chloroflexi and Planctomycetes. The abundance of Sphingobacteria was 22.9% in the partial nitritation process but was lower (4.2%) in the complete nitrification process, and Sphingobacteria have the function of removing nitrogen^15^. Similar phenomenon was also observed for the Chloroflexi (28.1% in partial nitritation process VS. 0.38% in complete nitrification process) and Planctomycetes (10.8% in partial nitritation process VS. 2.3% in complete nitrification process). It was noted that the Planctomycetes were present at a higher abundance in the partial nitritation process than in the complete nitrification process here. This was consistent with the previous study, where the Anammox was reported to be performed by the Planctomycetes^16,17^. In this study, the low DO concentration and co-existence of the nitrite and ammonia in the partial nitritation process provided a preferable condition for the growth of Planctomycetes, therefore resulting in the prevailing of Planctomycetes in the partial nitritation rather than the complete nitrification process.

In addition, another 4 groups of bacteria were found to have higher abundance in the complete nitrification process over the partial nitritation process, which included Alpha-proteobacteria, Delta-proteobacteria, Gamma-proteobacteria and Chlorobea. The abundances of these bacteria varied in a range of 10–13.8% in the complete nitrification process. Nevertheless, they became less abundant bacteria in the partial nitritation process, where the abundances were determined in a range of 0.8–4.8%. Especially, the nitrite oxidizing bacteria had a much higher abundance in the complete nitrification process (18%) relative to the partial nitritation process (2%), indicating the growth of the nitrite oxidizing bacteria was inhibited in the partial nitritation process. This was expected as the partial nitritation system in this study is an oxygen-scarce environment, which is not suitable for the growth of nitrite oxidizing bacteria^18^. This is also an indication that the operating conditions used in this study may facilitate the partial nitritation process. In this study, the dominant NOB were *Nitrobacter* sp. in the partial nitritation process, while both *Nitrobacter* sp. and *Nitrospira* sp. were detected as the dominant NOB in the nitritation step of complete nitrification process. It was reported that the *Nitrobacter sp*. have low affinity of substrates and high growth rates, whereas *Nitrospira sp*. have high affinity for substrates and low growth rate^19^. Therefore, the difference in the NOB composition may be due to the distinct concentration of nitrite between the two processes.

**Figure 7:**
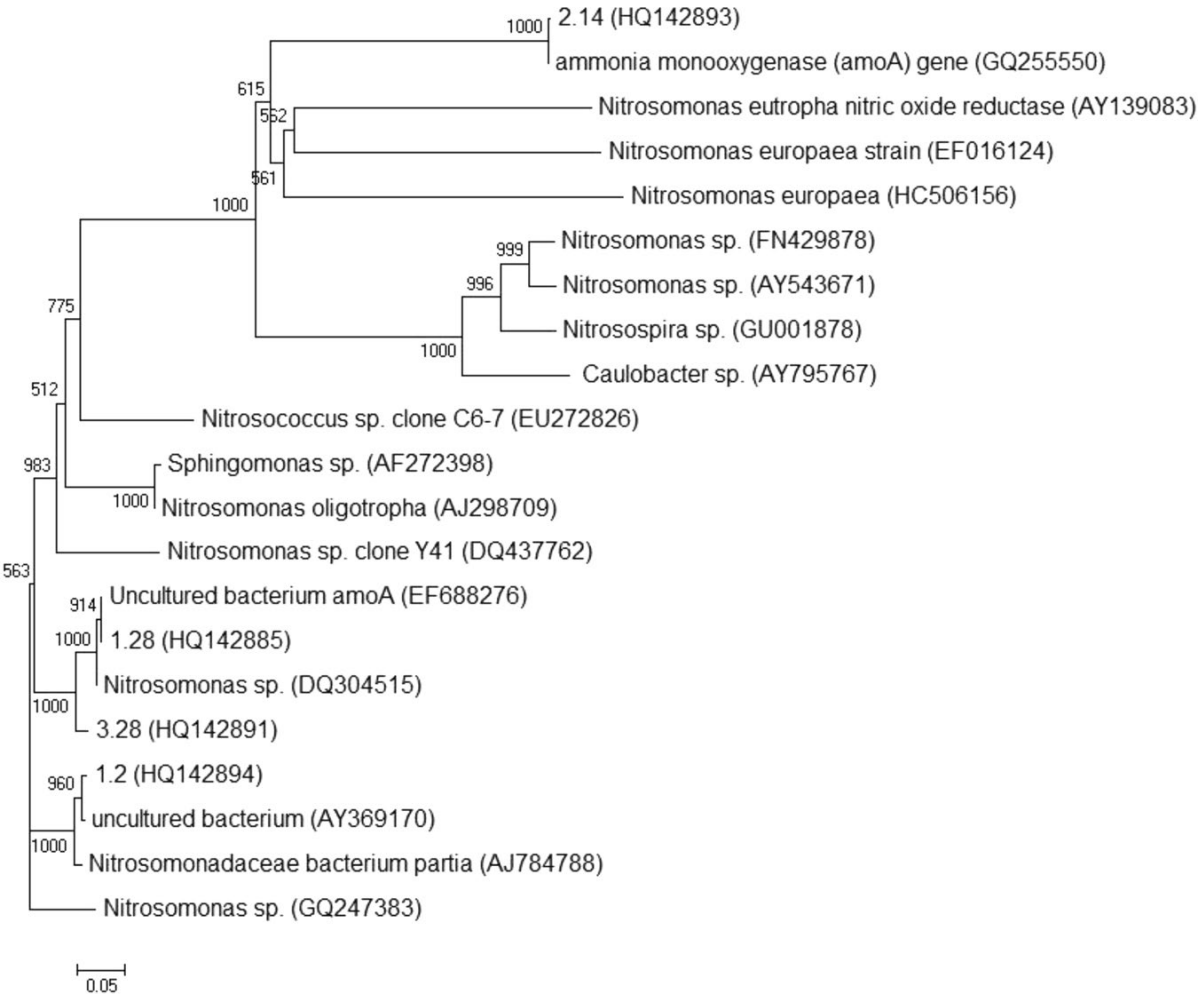
The phylogenetic tree of *amo*A gene sequences in the partial nitritation process. The construction of the phylogenetic tree was based on the Neighbour-joining method. The scale bar indicates two changes per 1000 nucleic acid positions.

An *amo*A gene clone library was constructed to specifically investigate the ammonia oxidizing bacteria in the partial nitritation process. 102 colonies were collected in the *amo*A clone library and categorized into 4 OTUs based on PCR-RFLP analysis. GenBank accession numbers for the 4 OTUs were HQ142893, HQ142885, HQ142891 and HQ142894. All 4 OTUs were detected as *Nitrosomonas* related microorganisms (Fig. 7). For example, The OUT 1.28, which was the most abundant OTU, was related to *Nitrosomonas* sp. (DQ304515) (99% *amo*A-based sequence similarity). These results further confirmed that the ammonia oxidization was mainly performed by the *Nitrosomonas* sp. in the partial nitritation process. It was reported that *amoA* gene could be used as a molecular marker to study the diversity of AOB^20^. This conclusion was supported by the results in this study. The dominant AOB indicated by *amoA* gene was represented by *Nitrosomonas* sp., which was consistent with the result from 16S rRNA in this and previous studies^14^.

## Conclusions

This study investigated the microbial community in the partial nitritation and complete nitrification processes. Our results from microbial analysis revealed that microbial community varied significantly with respect to process and operation conditions. In the partial nitritation process, the total microorganisms and ammonia oxidizing bacteria showed a high growth rate under the sludge circulating of 75%. The Beta-proteobacteria composed the most abundant bacterial groups of sludge in the both partial nitritation process and complete nitrification process. The *Nitrosomonas* sp. under Betaproteobacteria was the most dominant AOB group in the partial nitritation system. In comparison with the complete nitrification process, the growth of nitrite oxidizing bacteria, such as the *Nitrobacter* sp. and *Nitrospira* sp., was restricted in the partial nitritation process, indicating a good nitritation efficacy was acquired in this study. Further works may focus on the optimization of the operating parameters for partial nitritation process, in order to obtain a better nitrogen removal performance.

## Methods

### Experimental setup and operation

**Figure 8:**
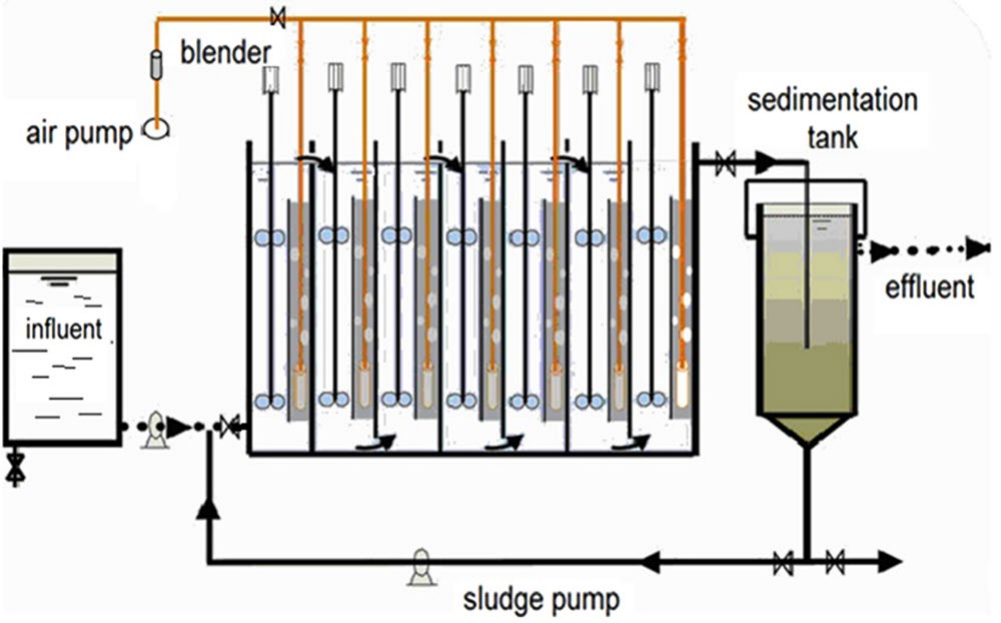
The schematic drawing of the baffled reactor used in this study.

A lab-scale baffled reactor (Fig. 8) was operated to treat the wastewater with low C/N ratio. The effective volume of the baffled reactor was 28 L. The baffled reactor was equally divided into 7 chambers by 6 baffles. In each chamber, an air-lift tube (2 cm in radius) was installed, which accounted for 15% of the volume of each chamber. Air sparging stones were installed at the bottom of the air-lift tubes. An aeration pump was used to provide oxygen to the reactor and to make the wastewater circulate continuously between the inside and outside of the air-lift tubes. A stirrer was installed in each chamber to mix the sludge biomass. The wastewater was collected from the Gaobeidian wastewater treatment plant in Beijing, China. The pH of the wastewater was approximately 7.5–8.0. The chemical oxygen demand (COD) and total ammonium of the wastewater was 23.4–92.8 mg/l and 43.0–54.7 mg/l respectively. The C/N ratio of the wastewater was in the range of 0.4–2.2. More information of the wastewater was in Table 1.

**Table 1:** The characteristics of wastewater used in the partial nitritation process and nitrification process.

Project	pH	COD (mg/l)	TN (mg/l)	NH_4_^+^-N (mg/l)	NO_2_^−^N (mg/l)	NO_3_^−^N (mg/l)	TP (mg/l)
Range	7.5–8.1	23.4–92.8	45.6–60.6	43.0–54.6	0–0.1	0.1–1.4	0.12–0.81
Average	7.7	51.5	51.5	49.2	0.05	0.7	0.36

When the baffled reactor was used in partial nitritation process, the concentration of dissolved oxygen (DO) out of the air-lift tubes was maintained below 0.2 mg/l. The flow rate of influent was maintained at 6 L·h^−1^ by a peristaltic pump. The hydraulic retention time was 4.6 h. The sludge circulating ratio was set from 25% to 100%, and each treatment was run for 30 days. The effluent of reactor were collected from the the last chamber at the circulating ratios of 25%, 50%, 75% and 100%, respectively. When the baffled reactor was used in the complete nitrification process, the concentration of DO in first two compartments was maintained at anoxic level (0.1–0.2 mg/l), and the aeration port is closed and DO was controlled by the rotor speed, respectively, while DO in last five compartments were maintained at aerobic level (3–4 mg/l), and the concentration of DO was controlled by the barometer.

### Phospholipid fatty acid analysis

In the partial nitritation process, the microbial community structures of the sludge samples under different circulating ratios, 25%, 50%, 75% and 100%, were determined by the phospholipid fatty acid (PLFA) analysis. The total lipid fractions were extracted based on the procedure described by Bligh and Dyer^21^. All solvents and chemicals used were of analytical grade. The lower lipid phase was removed and dried under a N_2_ stream_._ The lipid fraction containing the phospholipids was isolated and converted into fatty acid methyl esters based on a mild alkaline methanolysis reaction. Fatty acid methyl esters were analysed by a Hewlett-Packard 6890 gas chromatograph-HP5973 mass spectrometer (GC–MS) equipped with an HP-5 capillary column (60 m × 0.32 mm). Nonacosane acid methyl ester (19:0) was used as a quantitative internal standard. The quantities of the fatty acids were determined through comparing the peak areas with those of the standard peak. Fatty acid terminology utilizes “A:BωC,” where “A” indicates the total number of carbon atoms, “B” is the number of unsaturated carbons, and “ω” precedes “C,” means the number of C atoms between the closest unsaturated atom and the aliphatic end of the molecule. The prefixes *a* and *i* refer to anti-*iso* and *iso* methyl branching. The suffixes *c* and *t* indicate the *cis* and *trans* geometric isomers. Cyclopropyl groups are denoted by *cy*. The *9Me* refers to a methyl group on the ninth carbon from the carboxylic end of the fatty acid. Monounsaturated and branched-chain fatty acids were chosen to represent the PLFA of the bacterial group. The unsaturated PLFA 18:2ω6c and 18:3ω6c are used to represent fungal biomass.

### DNA extraction

Total genomic DNA of activated sludge was extracted from 0.5 g of sludge sample using a genomic DNA extraction kit (Omega, USA). The electrophoretic profile of the DNA products was determined using a 1% agarose gel. The concentrations of the DNA samples were quantified in Nanodrop ND-2000 (Thermo Technologies).

### Real-time quantitative PCR assay

Real-time quantitative PCR (qPCR) was performed on an Mx3000P fluorescent quantitative PCR thermocycler (Genetimes). Amplification reactions were performed with the SYBR Green PCR master mix (Tiangen, China). The PCR was performed in a 25 µl reaction system, including 12.5 µL PCR Master Mix, 2 µl forward primer (10 µM), 2 µl reverse primer (10 µm), 1 µl DNA template and 7.5 µl MilliQ water. The primers of CTO-189F/CTO-654R and NSR 1113 F/NSR 1264 R were selected to target the AOB 16S rRNA and NOB 16S rRNA, respectively (Table 2)^11^. The procedure of the qPCR included 1 cycle of predenaturation at 95 °C for 10 min, 40 cycles of denaturation step at 95 °C for 45 sec, annealing step at 56 °C for 45 s and extension step at 72 °C for 1 min^22^. The melting curves were routinely checked to confirm the purity of the amplified products. The parameter Ct (threshold cycle) was determined as the cycle number at which a statistically significant increase in reporter fluorescence was detected. The standard curves for real-time PCR assays were constructed using the 16S rRNA of *Nitrosomonas europaea* ATCC 19718 (AOB) and *Nitrobacter winogradskyi* ATCC 25391 (NOB)^23^. The negative control was the pure water. Briefly, AOB and NOB 16S rRNA genes were PCR-amplified from extracted DNA with the primers of CTO-189F/CTO-654R and NSR1113F/NSR1264R, respectively. The PCR products were cloned into the pGEM-T Easy Vector (Promega). The combined Plasmids containing PCR products were extracted to determine the copy numbers of the target genes and were used to construct the standard qPCR curves.

**Table 2:** Primers used for the PCR amplification.

Name	Sequence (5′-3′)	Target group
27F	AGAGTTTGATCCTGGCTCAG	16S rRNA
1492R	TACCTTGTTACGACTT	16S rRNA
*amo*A-1F	GGGGTTTCTACTGGTGGT	*amo*A gene
*amo*A-2R	CCCCTCKGSAAAGCCTTCTTC	*amo*A gene
CTO-189F	GGAGMAAAGYAGGGGATCG	AOB
CTO-654R	CTAGCYTTGTAGTTTCAAACGC	AOB
NSR 1113F	CCTGCTTTCAGTTGCTACCG	NOB
NSR 1264R	GTTTGCAGCGCTTTGTACCG	NOB

### Construction of clone library

The microbial community was studied by constructing the 16S rRNA clone libraries. The 16S rRNA genes of the sludge bacterial community were amplified by PCR using the universal primers 27F and 1492R (Table 2)^24^. The *amoA* genes of AOB were amplified with the primers *amoA* 1F and *amoA* 2R (Table 2)^25^. The plasmids of randomly selected positive colonies were re-amplified by PCR. The re-amplified PCR products were digested by the restriction endonucleases *Rsa* I and *Hha* I at 37 °C overnight to check the restriction fragment length polymorphism (RFLP) genetic profiles. In this step, the PCR products were clustered into different OTUs. The PCR product of each OTU was sequenced by the Sanger sequencing method (Beijing Huada Genes Bio-Tech Company, China).

### Sequences BLAST and Phylogenetic analysis

The DNA sequences were assigned to taxonomy against NCBI reference database using Nucleotide BLAST, and nucleotide sequences were entered into BLASTN programs nucleotide database query, that database selection other, optimize for Highly similar sequences, at last selected BLAST. The alignment of the sequences was performed in Clustal X 1.83. The phylogenetic tree was constructed by MEGA (version 4.1) based on the neighbour joining method, the scale bar indicates two changes per 1000 nucleic acid positions.

## Data Availability

The authors declare that all data are available and all data has been provided within the manuscript.
